# The effect of glucose-dependent insulinotropic polypeptide (*GIP*) variants on visceral fat accumulation in Han Chinese populations

**DOI:** 10.1038/nutd.2017.28

**Published:** 2017-05-22

**Authors:** T Wang, X Ma, T Tang, K Higuchi, D Peng, R Zhang, M Chen, J Yan, S Wang, D Yan, Z He, F Jiang, Y Bao, W Jia, K Ishida, C Hu

**Affiliations:** 1 Shanghai Diabetes Institute, Shanghai Key Laboratory of Diabetes Mellitus, Shanghai Clinical Center for Diabetes, Shanghai Jiao Tong University Affiliated Sixth People’s Hospital, Shanghai, China; 2 Laboratory of Cardiovascular Immunology, Institute of Cardiology, Union Hospital, Tongji Medical College of Huazhong University of Science and Technology, Wuhan, China; 3 Kao (China) Research & Development Center Company Limited, Shanghai, China; 4 Institute for Metabolic Diseases, Department of Endocrinology, Shanghai Jiao Tong University Affiliated Sixth People’s Hospital South Campus, Shanghai, China

## Abstract

**Objectives:**

We aim to validate the effects of glucose-dependent insulinotropic polypeptide (GIP) on fat distribution and glucose metabolism in Han Chinese populations.

**Methods:**

We genotyped six tag single-nucleotide polymorphisms (SNPs) of *GIP* and four tag SNPs of glucose-dependent insulinotropic polypeptide receptor (*GIPR*) among 2884 community-based individuals from Han Chinese populations. Linear analysis was applied to test the associations of these variants with visceral fat area (VFA) and subcutaneous fat area (SFA) quantified by magnetic resonance imaging as well as glucose-related traits.

**Results:**

We found that the C allele of rs4794008 of *GIP* tended to increase the VFA and the VFA/SFA ratio in all subjects (*P*=0.050 and *P*=0.054, respectively), and rs4794008 was associated with the VFA/SFA ratio in males (*P*=0.041) after adjusting for the BMI. The VFA-increasing allele of rs4794008 was not related to any glucose metabolism traits. However, rs9904288 of *GIP* was associated with the SFA in males as well as glucose-related traits in all subjects (*P* range, 0.004–0.049), and the *GIPR* variants displayed associations with both fat- and glucose-related traits.

**Conclusions:**

The results could provide the evidence that GIP might modulate visceral fat accumulation via incretin function or independent of incretin.

## Introduction

Obesity is currently one of the most common and severe complex disorders worldwide. It causes a great economic burden on public health not only due to the large number of individuals with obesity, but also the associated consequences.^1^ Visceral fat accumulation is the culprit in a variety of obesity-related disorders, including type 2 diabetes mellitus (T2DM), metabolic syndrome and cardiovascular diseases.^2^ Effective management and intervention for obesity, especially for visceral adiposity, should be implemented to decrease the prevalence of T2DM and other metabolic diseases.

Glucose-dependent insulinotropic polypeptide (GIP) is an important amino-acid peptide hormone that is secreted from the gut and then binds to glucose-dependent insulinotropic polypeptide receptors (GIPRs) after a meal. Given that GIPRs are expressed in various tissues, including pancreatic islets, adipocytes, brain and stomach, GIP signaling has been implicated in various activities, which may link overnutrition to obesity, insulin resistance and T2DM. Rodent studies have demonstrated the interference in the stimulation of glucose-stimulated insulin secretion as well as the modulation of beta-cell neogenesis, differentiation and proliferation (the so-called intrapancreatic actions).^3, 4, 5^ GIP also has additional extrapancreatic actions in addition to facilitating insulin release. The *vitro* studies on direct adipocyte actions showed GIP could enhance the lipoprotein lipase enzyme activity in cultured 3T3-L1 adipocytes.^6^ The blockade of GIPR signaling by GIPR knockout mice or GIP antagonist could decrease fat deposition under overnutrition.^7, 8, 9^ In support of this, increasing genetic evidence has demonstrated that *GIPR* variants were associated with plasma glucose levels,^10^ an index of incretin effect derived from an oral glucose tolerance test and an intravenous glucose tolerance test^11^ as well as with BMI^12, 13^ among European and East Asian populations, indicating that GIP signaling may participate in glucose metabolism and obesity. Nonetheless, the *GIP* variants directly linked to obesity or insulin dysfunction are less well characterized. Moreover, a recent study by Moller *et al.*^14^ indicated an association of the GIP level with low density lipoprotein and increased visceral fat area (VFA) independent of insulin action, suggesting the role of GIP in modulating adiposity deposits. Only one study from Japan showed that *GIP* variants might contribute to visceral fat accumulation as well as plasma triglyceride and hemoglobin A1c (HBA1c) levels irrespective of BMI.^15^

To increase our understanding of the contributions of the GIP–GIPR axis, our study aims to examine the effects of *GIP* and *GIPR* variants on fat distribution and metabolic traits among 2884 community-based individuals of Han Chinese ancestry.

## Materials and methods

### Subjects

Our study was approved by the institution review board of Shanghai Jiao Tong University Affiliated Sixth People’s Hospital in accordance with the principles of the Helsinki Declaration II. A total of 2884 community-based Han Chinese individuals were enrolled. Individuals with cancers, severe mental disorders and disabilities were excluded, and the remaining subjects provided written informed consent. The subjects received anthropometric measurements, magnetic resonance imaging assessment and laboratory examinations.

### Clinical phenotypes

Anthropometric measurements included height, weight and waist circumference (WC) and hip circumferences. The BMI was calculated as weight (kg)/height^2^ (m^2^), and the waist-to-hip ratio was calculated as the WC (cm)/hip circumference (cm) ratio. Body fat percentage (%) was assessed with a TBF-410 Tanita Body Composition Analyzer (Tanita, Tokyo, Japan). An abdominal magnetic resonance imaging scan was performed on each subject at the umbilicus level between L4 and L5 in the supine position (Archive, Philips Medical System, Amsterdam, the Netherlands). To calculate VFA (cm^2^) and subcutaneous fat area (SFA (cm^2^)), two trained observers assessed the images with SLICE-O-MATIC image analysis software (version 4.2; Tom Vision Inc., Montreal, QC, Canada). If the results differed by more than 10%, a third observer who was blinded to the results reanalyzed the images. Venous blood samples were drawn at 0, 30 and 120 min following glucose solution ingestion to assess glucose and insulin concentrations. Glucose levels were assayed using the glucose oxidase method, and insulin levels were measured using a radioimmunoassay (Linco Research, St Charles, MO, USA). We calculated the areas under the curve for glucose and insulin (G_AUC_ and I_AUC_) using the trapezoidal rule and estimated the insulinogenic index (change in insulin level/change in glucose level from 0 to 30 min). Insulin sensitivity and secretion were estimated according to the computations proposed by Stumvoll *et al.* and Gutt *et al.*,^16, 17^ and three indices were generated (Stumvoll first phase and second phase insulin secretion and the Gutt index).

### Tag SNP selection and genotyping

According to Nakayama *et al.*,^15^ six tag single-nucleotide polymorphisms (SNPs) of *GIP* located between 30 kb upstream and 30 kb downstream of the *GIP* region were selected based on the HapMap Phase III JPT+CHB database using a threshold of *r*^2^⩾0.8. We also selected four tag SNPs for *GIPR* that were located between 8 kb upstream and 24 kb downstream of the *GIPR* gene. These SNPs tag 100% of common SNPs with a minor allele frequency of >0.05. All of the SNPs were genotyped with a MassARRAY Compact Analyzer (Sequenom, San Diego, CA, USA). All of the SNPs passed quality control with call rates >95% and concordant rates >99%.

### Statistical analysis

The Hardy–Weinberg equilibrium was applied before analysis. Pairwise linkage disequilibrium analyses were performed using Haploview (version 4.2; www.broad.mit.edu/mpg/haploview/). The skewed distribution traits were log_10_-transformed. Linear regression analysis was used to test for the effects of SNPs on quantitative traits under the additive genetic model using PLINK (http://pngu.mgh.harvard.edu/~purcell/plink/). The analyses were adjusted for covariates, such as age, sex and other variables, if appropriate. Logistic regression was used to confirm the best model of PLINK epistatic analyses. A two-tailed *P*-value of <0.05 was considered significant.

Power calculations were performed by Quanto software (http://biostats.usc.edu/Quanto.html, version 1.2.4, May 2009) with current sample size. We had 71.6% power to detect an association for a SNP (minor allele frequency=0.46) accounting for 2.8 cm^2^ of the variation in VFA and 46.9% power to detect an association for this SNP accounting for 3.3 cm^2^ of the variation in SFA at the 0.05 significance.

## Results

### Association with obesity-related traits

All of the variants conformed to the Hardy–Weinberg equilibrium (*P*>0.05). The pairwise linkage disequilibrium maps of *GIP* and *GIPR* variants are shown in Supplementary Figure 1, and the subject characteristics are shown in Table 1.

As shown in Table 2, we observed that SNPs in *GIP* exhibited only borderline associations with fat distribution indices, including VFA, SFA and the VFA/SFA ratio. Specifically, rs11650936 was associated with the VFA/SFA ratio before adjusting for BMI (*P*=0.048), whereas the C allele of rs4794008 also tended to be associated with an increased VFA and VFA/SFA ratio after adjusting for BMI (*P*=0.050 and *P*=0.054, respectively). In contrast, minor associations between rs11671664 in *GIPR* were observed for VFA and SFA before adjusting for BMI (*P*=0.018 and *P*=0.020, respectively). The previously reported *GIPR* SNP rs11671664 was related to BMI and WC as expected, whereas rs12941604 of *GIP* showed a slight association with WC (*P* range, 0.0043–0.0184).

Because of the heterogeneity in adiposity function and adipose deposits between males and females, we also performed a subgroup analysis stratified by gender (shown in Table 3). rs4794008 of *GIP* was associated with the VFA/SFA ratio without or with adjustment of the BMI in male subjects (*P*=0.040 and *P*=0.041, respectively). The other two SNPs, rs9904288 and rs2291725, of *GIP* were associated with SFA after adjustment of the BMI in males (*P*=0.004 and *P*=0.029, respectively). Regarding the *GIPR* SNPs, rs11671664 was associated with VFA before BMI adjustment in males (*P*=0.030). In contrast, only rs11671664 of *GIPR* showed a slight association with SFA in female subjects (*P*=0.049). However, we failed to identify any gender interaction of these variants for distribution traits.

### Association with glucose metabolism traits

In terms of glucose-related traits, rs9904288 of *GIP* was the most significant SNP among *GIP* variants and was associated with a range of glucose metabolism traits (shown in Table 4). The SFA-increasing allele rs9904288 was associated with decreased 2 h glucose and 2 h insulin levels and an elevated insulinogenic index and insulin sensitivity (assessed with the Gutt index) (*P* range, 0.014–0.049), whereas the rs4794008 SNP of *GIP* showed a nominal association with VFA in males and no association with glucose-related traits. Compared to the tag SNPs of *GIP*, rs2287019 and rs11671664 of *GIPR* were associated with the glucose and insulin levels, the insulinogenic index and the Gutt index (*P* range, 9.46 × 10^−5^–0.028).

### Gene–gene epistasis

**Figure 1:**
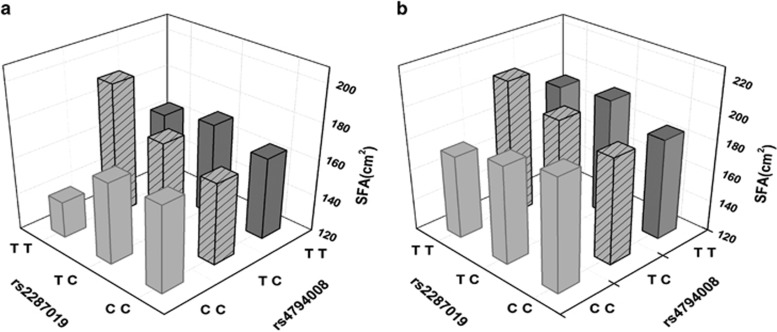
Epistatic analysis between rs4794008 of GIP and rs2287019 of GIPR in subcutaneous fat. The SFA values for each genotype of rs4794008 of *GIP* and rs2287019 of *GIPR* represent the mean values in total subjects (**a**) (*n*=2884) and in female subjects (**b**) (*n*=1562).

To avoid overlooking the heritability of obesity traits due to unknown interactions between *GIP* and *GIPR* variants, we performed gene–gene interaction (epistasis) analyses. The results showed that rs4794008 of *GIP* and rs2287019 of *GIPR* exhibited significant epistatic effects on SFA in all subjects and in female subjects using the genotypic model (*P*=0.0313 and *P*=0.0178, respectively) (Figure 1).

## Discussion

We investigated the association of six tag SNPs of *GIP* and four tag SNPs of *GIPR* with fat distribution and glucose-related traits in 2884 Han Chinese individuals. rs4794008 of *GIP* was associated with visceral fat accumulation, whereas other *GIP* and *GIPR* variants (that is, rs9904288 of *GIP*, rs11671664 and rs2287019 of *GIPR*) were related to both fat distribution and glucose-related traits. Moreover, we found that rs4794008 of *GIP* and rs2287019 of *GIPR* exhibited significant epistatic effects on subcutaneous fat accumulation. Consistent with the physiological and pathological functions of the GIP–GIPR axis on intrapancreatic and extrapancreatic activity, our findings indicated that *GIP* variants could regulate visceral adiposity via two possible paths that were either mediated by incretin effects or independent of incretin effects.

Similar to previous genome-wide association study (GWAS) analyses between European and East Asian populations,^12, 13, 18^ we observed that rs11671664 and 2287019 of *GIPR* were associated with BMI and glucose-related traits. Nakayama *et al.*^15^ found that rs9904288 of *GIP* was related to visceral fat accumulation, but rs4794008 only displayed an association with the HBA1c level in Japanese populations, which we did not replicate in this study. Although the sample sizes were comparable, the heterogeneity between the two studies was expected based on the use of the bioelectrical impedance method in the Japanese study and magnetic resonance imaging scans in our study to assess visceral fat and subcutaneous fat accumulation. Moreover, to determine whether these associations with *GIP* variants reflected differences between overall obesity and fat distribution, we repeated our association analysis, including BMI, as a covariate. Studies that directly investigate the associations of *GIP* and *GIPR* variants with fat distribution and related metabolic traits independent of BMI must be conducted. Harada *et al.*^19^ identified that a splice *GIPR* variant expressed in mouse pancreatic cells affected GIPR sensitivity in high-fat diet-induced obese mice. Whether the variants of *GIPR* tested in our study impact the sensitivity of GIPR in human body needs to be investigated in further study.

A series of studies provided evidence supporting the role of GIP in regulating obesity. *In vitro* studies on direct adipocyte action indicated that GIP stimulates adipocyte lipoprotein lipase activity, which is responsible for the hydrolysis of triglycerides in circulating blood and for promoting lipogenesis by increasing free fatty acid uptake by adipocytes.^6, 20^ The effects of GIP on animal and human adipose storage and metabolism are mixed. Mice maintained on a high-fat diet exhibited increased *GIP* mRNA expression, GIP secretion and K-cell density, and inhibition of GIP action by *GIPR* ablation or antagonists reversed high-fat-induced obesity and improved insulin sensitivity^7, 9, 21^ Nonetheless, some other studies showed that GIPR^−/−^ mice exhibited similar adiposity with wild-type mice on normal diet^7^ and specific GIPR knockout mice in adiposity did not reduce fat volume but decreased liver weight and insulin resistance.^22^ GIP in combination with hyperinsulinemia and hyperglycemia increased triacylglycerol deposition in subcutaneous fat by enhancing free fatty acid re-esterification in lean human subjects.^23^ However, in obese patients, GIP did not induce changes in triacylglycerol uptake in adipose tissue during hyperinsulinemia and hyperglycemic clamping,^24^ potentially due to disrupted GIP signaling in insulin-resistant and excess weight states. A recent study pointed out that GIP infusion was able to stimulate insulin secretion in the lean, obesity or obesity patients with impaired glucose regulation (IGR) rather than obesity patients with T2DM, whereas resulted in the anabolic effect (that means exaggerated fat deposit) in obesity patients with T2DM, indicating the blunted insulinotropic but preserved lipogenic actions in obesity patients with T2DM.^25^ To date, it is difficult to dissect the separate contributions of insulin and GIP to glucose and lipid metabolism. We primarily identified *GIP* variants that likely regulate visceral adiposity via two possible paths: mediated by incretin or independent of incretin effects. The pattern by which genetic variants interact as well as the modifying role of insulin appears different between normal individuals and individuals with obesity, which can be informative about biological function in further.

The concept of epistasis originally referred to an allelic effect at one locus being concealed by the effect of another allele at a second locus. However, a more recent definition has been extended to include the effect of an allele at a genetic variant that depends either on the presence or absence of another genetic variant.^26^ Considering the biological crosstalk between GIP and GIPR, we searched for epistatic effects. Interestingly, although rs4794008 of *GIP* and rs2287019 of *GIPR* were not associated with SFA *per se*, they exhibited statistically significant epistatic effects on subcutaneous fat accumulation in all subjects and in female subjects. The discovery and replication of functional epistasis are warranted in the interpretation of genetic association studies.

Several limitations of our study should be noted. First, the cross-sectional nature of the study prevented us from investigating the effect of *GIP* variants on the natural history of visceral fat accumulation. Second, all of the variants tested in our study were in non-coding regions, and the potential relationship between GIP levels, fat distribution and related metabolic traits should be investigated in future studies. Moreover, false positives should not be excluded due to the modest effect of *GIP* variants on fat distribution traits and the difficulty of performing multiple corrections. Nonetheless, our findings provide novel information based on previous functional evidence implying that the possible paths by which *GIP* variants modulate visceral fat accumulation can be incretin dependent or independent. Thus, it is imperative to replicate the effect of *GIP* variants on visceral fat accumulation and related metabolic traits.

## Conclusions

In summary, we observed that *GIP* rs4794008 was associated with visceral fat accumulation, and other *GIP* and *GIPR* variants were related to both fat distribution and glucose-related traits in 2884 Han Chinese individuals, implying that *GIP* variants regulate visceral adiposity via incretin-dependent and -independent effects. Further functional studies are needed to confirm and elucidate the underlying mechanism and the characteristics of treatment responses to become a potential target for obesity.

## Figures and Tables

**Table 1:** Subject characteristics

	*Overall*	*Males*	*Females*
N	2884	1322	1562
AGE (year)	52.05±6.93	52.11±6.95	52.01±6.92
BMI (kg m^−2^)	24.44±3.36	24.90±3.21	24.05±3.43
WC (cm)	86.00 (80.00, 93.00)	88.50 (83.00, 95.00)	83.50 (77.50, 90.20)
WHR	0.91 (0.87, 0.96)	0.93 (0.90, 0.96)	0.89 (0.86, 0.94)
VFA (cm^2^)	75.75 (51.65, 108.96)	92.48 (63.60, 127.88)	65.64 (47.18, 89.45)
SFA (cm^2^)	157.27 (117.99, 205.21)	130.8 (102.3, 169.25)	184.05 (140.10, 230.35)
VFA/SFA ratio	0.47 (0.32, 0.69)	0.67 (0.50, 0.90)	0.35 (0.26, 0.47)
Fasting glucose (mmol l^−1^)	5.22 (4.79, 5.80)	5.33 (4.89, 6.00)	5.17 (4.72, 5.60)
2 h glucose (mmol l^−1^)	6.00 (4.89, 7.66)	6.00 (4.70, 8.11)	6.00 (4.96, 7.40)
Fasting insulin (mU l^−1^)	9.40 (7.00, 12.64)	9.36 (6.86, 12.63)	9.42 (7.17, 12.64)
2 h insulin (mU l^−1^)	31.88 (20.08, 49.59)	28.68 (16.88, 46.99)	34.63 (22.72, 51.72)
G_AUC_	14.63 (12.35, 17.39)	15.27 (12.93, 18.41)	14.14 (11.92, 16.63)
I_AUC_	70.40 (49.04, 100.28)	67.34 (45.69, 98.97)	72.27 (52.54, 101.07)
Insulinogenic index (mU mmol^−1^)	9.81 (5.21, 17.49)	8.20 (4.17, 15.36)	11.08 (6.17, 19.19)
Stumvoll first phase insulin secretion (pmol l^−1^)	934.21 (647.08, 1253.65)	864.29 (556.12, 1223.07)	983.01 (723.02, 1273.25)
Stumvoll second phase insulin secretion (pmol l^−1^)	253.52 (190.00, 323.65)	236.51 (169.91, 318.30)	263.66 (206.69, 327.99)
Gutt index	82.82 (62.99, 106.79)	83.26 (60.37, 111.81)	82.33 (64.14, 102.69)

Abbreviations: G_AUC_, area under the curve for glucose; I_AUC_, area under the curve for glucose; VFA, visceral fat area; VFA/SFA, the ratio of visceral fat to subcutaneous fat; WC, waist circumfference; WHR, waist-to-hip ratio.

Data are shown as mean+s.d., median (interquartile range) or *N* (%).

**Table 2:** Association with obesity-related traits

*SNP*	*Gene*	*Chr*	*Position*	*Allelle*	*MAF*	*Traits*	*Model 1*			*Model 2*		
							β	*s.e.*	P	β	*s.e.*	P
rs12941604	*GIP*	17	5E+07	A/G	0.09	VFA	0.01	0.011	0.367	−0.003	0.008	0.688
						SFA	0.011	0.008	0.175	0	0.005	0.954
						VFA/SFA	−0.001	0.009	0.954	−0.004	0.009	0.669
rs9904288	*GIP*	17	5E+07	C/T	0.17	VFA	−0.005	0.008	0.553	0.001	0.006	0.916
						SFA	0.003	0.006	0.57	0.008	0.004	0.057
						VFA/SFA	−0.008	0.007	0.213	−0.007	0.007	0.278
rs2291725	*GIP*	17	5E+07	T/C	0.29	VFA	−0.005	0.007	0.439	−0.003	0.005	0.554
						SFA	0.002	0.005	0.626	0.004	0.003	0.237
						VFA/SFA	−0.008	0.005	0.161	−0.007	0.005	0.188
rs4794008	*GIP*	17	5E+07	T/C	0.25	VFA	−0.009	0.007	0.23	−0.011	0.005	0.05
						SFA	0.002	0.005	0.733	0	0.004	0.998
						VFA/SFA	−0.01	0.006	0.067	−0.011	0.006	0.054
rs1390154	*GIP*	17	5E+07	T/C	0.38	VFA	0.002	0.006	0.707	−0.001	0.005	0.81
						SFA	0.003	0.005	0.532	0	0.003	1
						VFA/SFA	0	0.005	0.94	−0.001	0.005	0.816
rs11650936	*GIP*	17	5E+07	G/C	0.17	VFA	−0.009	0.009	0.276	−0.007	0.006	0.285
						SFA	0.004	0.006	0.508	0.006	0.004	0.182
						VFA/SFA	−0.013	0.007	**0.048**	−0.013	0.007	0.057
rs11671664	*GIPR*	19	5E+07	A/G	0.46	VFA	−0.015	0.006	**0.018**	−0.003	0.005	0.502
						SFA	−0.01	0.004	**0.02**	−0.001	0.003	0.68
						VFA/SFA	−0.005	0.005	0.341	−0.002	0.005	0.677
rs13306402	*GIPR*	19	5E+07	T/C	0.01	VFA	0.024	0.041	0.557	0.022	0.031	0.485
						SFA	−0.015	0.029	0.601	−0.017	0.02	0.404
						VFA/SFA	0.039	0.032	0.223	0.039	0.032	0.222
rs2334255	*GIPR*	19	5E+07	T/G	0.46	VFA	−0.005	0.006	0.426	−0.001	0.005	0.844
						SFA	−0.001	0.004	0.751	0.002	0.003	0.575
						VFA/SFA	−0.004	0.005	0.466	−0.003	0.005	0.584
rs2287019	*GIPR*	19	5E+07	T/C	0.18	VFA	0	0.008	0.956	0.001	0.006	0.857
						SFA	0.001	0.006	0.919	0.002	0.004	0.635
						VFA/SFA	−0.001	0.007	0.871	−0.001	0.006	0.9

Abbreviations: Allele, minor/major allele; MAF, minor allele frequency; s.e., standard error; SFA, subcutaneous fat area; SNP, single-nucleotide polymorphism; VFA, visceral fat area; VFA/SFA, the ratio of visceral fat to subcutaneous fat.

*P* values <0.05 are shown in bold.

Traits were adjusted for age and sex in the additive genetic model 1 and adjusted for age, sex and BMI in model 2.

**Table 3:** Gender differences in how the variants influence fat distribution

*SNP*	*Gene*	*Chr*	*Position*	*Allelle*	*MAF*	*Traits*	*Males*				*Females*			
							β	*s.e.*	P^a^	P^b^	β	*s.e.*	P^a^	P^b^
rs12941604	*GIP*	17	48929651	A/G	0.09	VFA	0.006	0.018	0.757	0.98	0.016	0.013	0.23	0.827
						SFA	0.006	0.012	0.594	0.759	0.015	0.01	0.142	0.906
						VFA/SFA	−0.001	0.013	0.969	0.892	0.001	0.012	0.931	0.776
rs9904288	*GIP*	17	48954611	C/T	0.17	VFA	−0.017	0.014	0.206	0.73	0.007	0.01	0.508	0.747
						SFA	0	0.009	0.994	**0.004**	0.007	0.008	0.39	0.538
						VFA/SFA	−0.017	0.01	0.081	0.198	0	0.009	0.98	0.916
rs2291725	*GIP*	17	48961770	T/C	0.29	VFA	−0.015	0.011	0.195	0.825	0.004	0.008	0.651	0.908
						SFA	0	0.007	0.977	**0.029**	0.005	0.006	0.456	0.823
						VFA/SFA	−0.015	0.008	0.072	0.141	−0.001	0.007	0.896	0.803
rs4794008	*GIP*	17	48971599	T/C	0.25	VFA	−0.016	0.012	0.174	0.118	−0.002	0.009	0.833	0.316
						SFA	0.002	0.008	0.843	0.495	0.002	0.007	0.737	0.726
						VFA/SFA	−0.018	0.009	**0.04**	**0.041**	−0.004	0.007	0.59	0.508
rs1390154	*GIP*	17	48994121	T/C	0.38	VFA	0.003	0.01	0.74	0.771	0.002	0.008	0.832	0.572
						SFA	0.003	0.007	0.7	0.687	0.003	0.006	0.613	0.827
						VFA/SFA	0.001	0.008	0.918	0.946	−0.001	0.007	0.849	0.728
rs11650936	*GIP*	17	48994899	G/C	0.17	VFA	−0.002	0.014	0.871	0.547	−0.012	0.01	0.264	0.455
						SFA	0.01	0.009	0.263	0.21	0	0.008	0.968	0.462
						VFA/SFA	−0.012	0.01	0.219	0.176	−0.012	0.009	0.179	0.24
rs11671664	*GIPR*	19	45669020	A/G	0.46	VFA	−0.022	0.01	**0.03**	0.183	−0.009	0.008	0.224	0.736
						SFA	−0.009	0.007	0.173	0.976	−0.012	0.006	**0.049**	0.534
						VFA/SFA	−0.013	0.007	0.076	0.152	0.002	0.007	0.753	0.48
rs13306402	*GIPR*	19	45674095	T/C	0.01	VFA	0.006	0.066	0.929	0.809	0.018	0.05	0.721	0.443
						SFA	−0.017	0.043	0.689	0.25	−0.022	0.038	0.562	0.646
						VFA/SFA	0.023	0.048	0.629	0.685	0.04	0.043	0.348	0.314
rs2334255	*GIPR*	19	45682892	T/G	0.46	VFA	−0.01	0.01	0.321	0.48	−0.002	0.008	0.779	0.777
						SFA	0.002	0.007	0.754	0.168	−0.005	0.006	0.39	0.634
						VFA/SFA	−0.012	0.007	0.097	0.122	0.003	0.007	0.654	0.557
rs2287019	*GIPR*	19	45698914	T/C	0.18	VFA	−0.002	0.013	0.87	0.6	0.002	0.01	0.812	0.377
						SFA	0.004	0.009	0.642	0.752	−0.002	0.008	0.802	0.77
						VFA/SFA	−0.006	0.01	0.52	0.464	0.004	0.009	0.623	0.556

Abbreviations: Allele, minor/major allele; MAF, minor allele frequency; *P*^*a*^, *P-*value adjusted age and sex; *P*^*b*^, *P-*value adjusted age, sex and BMI; s.e., standard error; SFA, subcutaneous fat area; SNP, single-nucleotide polymorphism; VFA, visceral fat area; VFA/SFA, the ratio of visceral fat to subcutaneous fat.

*P* values <0.05 are shown in bold.

**Table 4:** The associations with glucose metabolism traits

	*GIP_rs9904288*			*GIP_rs4794008*			*GIPR_rs2287019*			*GIPR_rs11671664*		
	β	*s.e.*	P	β	*s.e.*	P	β	*s.e.*	P	β	*s.e.*	P
Fasting glucose	−0.07	0.056	0.206	0.01	0.045	0.818	0.12	0.055	**0.028**	0.046	0.042	0.265
30 min glucose	−0.163	0.1	0.105	−0.007	0.084	0.932	0.152	0.099	0.127	0.196	0.075	**0.009**
2 h glucose	−0.256	0.13	**0.049**	0.06	0.109	0.581	0.496	0.128	**1.11 × 10**^**−4**^	0.155	0.097	0.112
Fasting insulin	−0.009	0.007	0.224	−0.008	0.006	0.205	0.015	0.007	**0.028**	−0.001	0.005	0.82
30 min insulin	0.008	0.01	0.393	−0.011	0.008	0.194	−0.028	0.01	**0.003**	−0.008	0.007	0.256
2 h insulin	−0.021	0.01	**0.035**	−0.01	0.009	0.24	0.01	0.01	0.331	−0.001	0.008	0.857
G_AUC_	−0.372	0.193	0.054	0.04	0.162	0.803	0.554	0.191	**0.004**	0.323	0.145	**0.025**
I_AUC_	−0.003	0.008	0.682	−0.011	0.007	0.102	−0.015	0.008	0.06	−0.006	0.006	0.345
Insulinogenic index	0.036	0.016	**0.027**	−0.014	0.014	0.329	−0.048	0.016	**0.003**	−0.048	0.012	**9.46 × 10**^**−5**^
Stumvoll first phase insulin secretion	0.002	0.011	0.865	−0.013	0.01	0.172	−0.018	0.011	0.114	−0.023	0.009	**0.007**
Stumvoll second phase insulin secretion	0	0.009	0.969	−0.005	0.007	0.52	−0.006	0.008	0.509	−0.015	0.006	**0.02**
Gutt index	0.015	0.006	**0.014**	0.003	0.005	0.616	−0.022	0.006	**2.51 × 10**^**−4**^	−0.005	0.005	0.229

Abbreviations: G_AUC_, area under the curve of the glucose from 0 to 120 min; I_AUC_, area under the curve of the insulin from 0 to 120 min; s.e., standard error.

Insulinogenic index, change of insulin levels/change of glucose levels from 0 to 30 min;

*P* values <0.05 are shown in bold.

Traits were adjusted for age, sex and BMI in the additive model.
