# Supplementary material for: The effect of glucose-dependent insulinotropic polypeptide (GIP) variants on visceral fat accumulation in Han Chinese populations
Source: Nutr Diabetes. 2017 May 22;7(5):e278–. doi: 10.1038/nutd.2017.28 (PMC5518809; doi:10.1038/nutd.2017.28)
Supplement: Supplementary Figure Legends [file nutd201728x1.docx]

**The Effect of Glucose-dependent Insulinotropic Polypeptide (*GIP)* Variants on Visceral Fat Accumulation in Han Chinese Populations**

Tao Wang ^a^, Xiaojing Ma ^a^, Tingting Tang ^b^, Kazuhiko Higuchi ^c^, Danfeng Peng ^a^, Rong Zhang ^a^, Miao Chen ^a^, Jing Yan ^a^, Shiyun Wang ^a^, Dandan Yan ^a^, Zhen He ^a^, Feng Jiang ^a^, Yuqian Bao ^a^, Weiping Jia ^a^, Koichi Ishida ^c^, Cheng Hu ^a,d,^*

**Supplementary Figure legends**

**Supplementary Figure 1. Linkage disequilibrium (LD) maps for SNPs genotyped in the *GIP* and *GIPR* region.** LD blocks of (A) *GIP* and (B) *GIPR* are shown. Each diamond represents the LD degree between the SNPs. The color indicates the r^2^ (a darker grey color represents a higher r^2^), and the numbers within the diamonds indicate the r^2^ values expressed as percentages.
