# Supplementary figures and images for: The effect of glucose-dependent insulinotropic polypeptide (GIP) variants on visceral fat accumulation in Han Chinese populations
Source: Nutr Diabetes. 2017 May 22;7(5):e278–. doi: 10.1038/nutd.2017.28 (PMC5518809; doi:10.1038/nutd.2017.28)

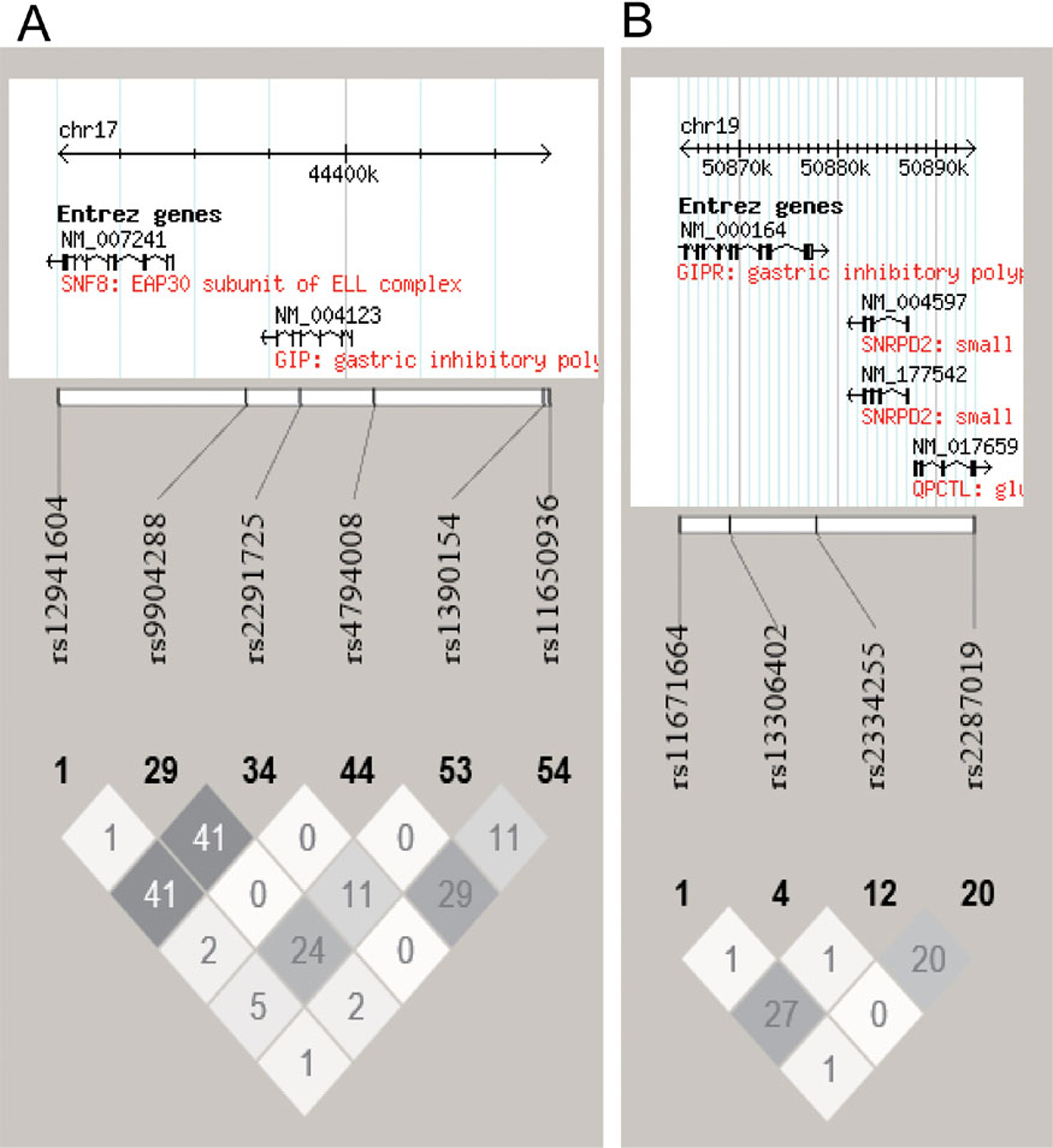

Supplement: Supplementary Figure 1 [file nutd201728x2.tif]
